# Docetaxel plus cisplatin is effective for patients with metastatic breast cancer resistant to previous anthracycline treatment: a phase II clinical trial

**DOI:** 10.1186/1471-2407-5-21

**Published:** 2005-02-22

**Authors:** Se Hoon Park, Eun Kyung Cho, Soo-Mee Bang, Dong Bok Shin, Jae Hoon Lee, Young Don Lee

**Affiliations:** 1 Division of Hematology and Oncology, Internal Medicine, Gachon Medical School Gil Medical Center, Incheon 405–760, Korea; 2 Department of Breast Surgery, Gachon Medical School Gil Medical Center, Incheon 405–760, Korea

## Abstract

**Background:**

Patients with metastatic breast cancer (MBC) are frequently exposed to high cumulative doses of anthracyclines and are at risk of resistance and cardiotoxicity. This phase II trial evaluated the efficacy and toxicity of docetaxel plus cisplatin, as salvage chemotherapy in patients with MBC resistant to prior anthracyclines.

**Methods:**

Patients with MBC that had progressed after at least one prior chemotherapy regimen containing anthracyclines received docetaxel 75 mg/m^2 ^followed by cisplatin 60 mg/m^2 ^every 3 weeks for a maximum of 6 cycles or until disease progression.

**Results:**

Between Jan 2000 and May 2002, 24 patients with tumors primary resistant and 15 with secondary resistant disease were accrued. All 39 patients were evaluable for safety and 36 for efficacy. The objective response rate was 31% (95% CI, 16–45%) with 3 complete responses. The median time to disease progression was 7 months, and the median overall survival was 23 months (median follow-up of 41 months). Neutropenia was the most frequently observed severe hematologic toxicity (39% of patients), whereas asthenia and nausea were the most common non-hematologic toxicities. No treatment-related death was observed.

**Conclusion:**

In conclusion, we found docetaxel plus cisplatin to be an active and safe chemotherapy regimen for patients with MBC resistant to anthracyclines.

## Background

In the management of breast cancer, anthracycline-based chemotherapy regimens remain standard adjuvant or first-line palliative treatment. Furthermore, some patients cannot be treated with anthracyclines due to impaired cardiac function. It is thus important to identify active, well-tolerated, not anthracycline cross-resistant, salvage regimens [1].

Taxanes (docetaxel and paclitaxel) are currently the most extensively studied new chemotherapeutic agents for metastatic breast cancer (MBC). Single-agent docetaxel has demonstrated significant survival advantages over other recognized regimens in 2 large randomized trials in patients with anthracycline-pretreated MBC [2,3]. Phase II data suggest that docetaxel is the most active agent yet available for the treatment of MBC [4]. Docetaxel also has some activity in paclitaxel-resistant MBC [5].

Cisplatin monotherapy has shown response rate of 9% in salvage settings, and 50% as first-line therapy [6]. Because docetaxel and cisplatin are both active and have different mechanisms of action, this combination may provide additive effect against MBC. Although docetaxel and cisplatin were not actively synergistic in preclinical studies [7,8], this combination chemotherapy has been widely used for treatment of a variety of tumor types [9-13]. Prior phase I studies showed the feasibility of this combination and its activity on different tumors [14]. Considering the single-agent activity of both drugs, their different mechanism of action and distinct toxicity profile, we designed this phase II study, in which the combination was evaluated as a salvage therapy in patients with anthracycline-resistant MBC.

## Methods

### Eligibility

Eligible patients had measurable or assessable histologically confirmed breast cancer that had progressed after at least one prior chemotherapy regimen containing anthracyclines. All patients had Eastern Cooperative Oncology Group performance status of 2 or lower and adequate bone marrow, hepatic, and renal function, defined as white blood cells ≥4000/μL, absolute neutrophil count ≥1500/μL, platelets ≥100,000/μL, total bilirubin ≤2.0 mg/dL, transaminases ≤3 times the upper normal limit, and serum creatinine ≤1.5 mg/dL. Concurrent radiation or hormonal therapy was not allowed; however, patients with clinically stable metastases of the brain or other sites who had completed radiation therapy were permitted. Patients were eligible regardless of the nature of prior therapy, including high-dose therapy with stem cell support and prior exposure to paclitaxel. Any prior antitumor treatment had been completed at least 1 month before entering this study. Patients were excluded if they had severe comorbid illness, symptomatic peripheral neuropathy of any origin, or a history of anaphylaxis of any type. All patients were provided a thorough explanation of the study, and they signed informed consent prior to enrollment into the study.

### Definition of anthracycline-resistance

Patients were classified as primary and secondary anthracycline-resistant. Primary anthracycline-resistance was defined as relapse during or within 12 months after anthracycline-based adjuvant therapy, or disease progression with no intervening response during anthracycline-containing chemotherapy for MBC. Secondary resistance was defined as relapse more than 12 months after receiving adjuvant anthracycline chemotherapy, or disease progression at some time after a documented clinical response to anthracycline-based chemotherapy for MBC.

### Pretreatment and follow-up evaluation

Pretreatment evaluation included a complete medical history and physical examination, a computed tomographic (CT) scan of the chest and abdomen, magnetic resonance imaging (MRI) of the brain, and a bone scintigraphy to assess the extent of disease. Follow-up consisted of physical examination, monitoring of toxic effects, a complete blood count, liver function tests, chest radiography, and CT scan as clinically indicated.

Tumor response and toxicity were recorded in accordance with the World Health Organization (WHO) criteria. Responses were assessed every 2 cycles of chemotherapy, and patients were evaluated before each new treatment cycle for toxicities.

### Treatment plan

The treatment consisted of docetaxel (Taxotere^®^, Aventis, Bridgewater, NJ) 75 mg/m^2 ^given by a 1-hour intravenous infusion immediately followed by cisplatin 60 mg/m^2 ^in a 2-hour infusion. Cycles were repeated every 3 weeks if the patient's blood count had returned to normal and non-hematologic toxicities had resolved. Dosage of subsequent cycles was adjusted according to the observed toxicities that developed during the preceding cycle. The treatment continued for a maximum of 6 cycles or until disease progression. All patients received standard supportive care regimen consisting of adequate hydration, dexamethasone, and antiemetic therapy according to the guidelines of the American Society of Clinical Oncology [15].

### Statistics

The primary end points of the trial were the efficacy of the therapy, which were measured as objective response rate and time to progression, with a secondary end point of toxicity. All patients who received at least 2 cycles of treatment or who progressed after the first cycle were considered assessable for response. The study was conducted using a single-stage, phase II trial design [16]. Based on the results from previous phase II trials reporting response rates of about 30% in patients with anthracycline-resistant MBC, we selected a 50% target response probability and a 30% null response probability, with type I and type II error set at 10%. The number of patients required was 39.

Statistical calculations were performed using SPSS software, version 11.5 (SPSS, Inc, Chicago, IL). Comparisons were performed using chi-square test and survival curves were generated using Kaplan-Meier method. Results were considered significant at the P = .05.

## Results

### Patients characteristics

Between Jan 2000 and May 2002, 24 patients with tumors primary resistant and 15 with secondary resistant disease were accrued. Among 39 patients assessable for safety, 3 were not evaluable for response due to early discontinuation of treatment.

Baseline characteristics of the eligible patients are listed in Table 1. Median age was 51 years (range, 41–64) and median performance status was 1. Thirty-three patients (85%) had visceral metastases as the dominant site of disease. All patients had received prior anthracycline-based chemotherapy with a median number of prior chemotherapeutic regimens of 2 (range, 1–3). Three patients (8%) had exposed to paclitaxel in their previous chemotherapy regimen.

### Efficacy

According to the "intent-to-treat" analysis, the objective response rate was 31% (95% confidence interval [CI], 16–45%). There were 3 complete responses, 12 partial responses and 15 patients had stable disease. The three patients who obtained a complete response had non-visceral metastatic pattern (neck lymph nodes and/or soft tissue lesions). Most patients who achieved a response did so at the end of the third cycle (median time to response, 2.2 months; 95% CI, 2.1–2.4 months).

**Figure 1:**
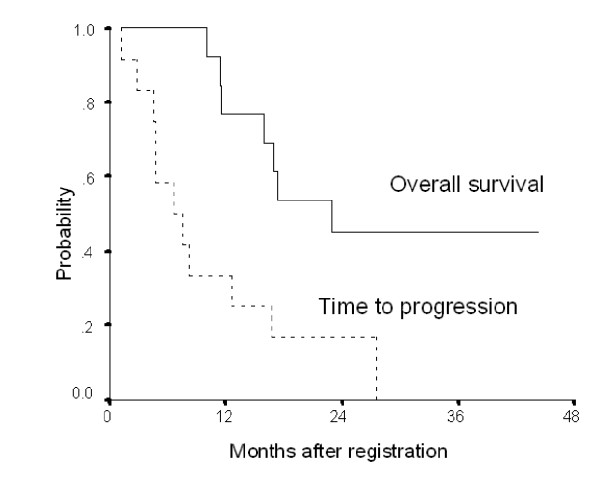
**Estimated curves of overall survival and time to progression**. At a median follow-up of 40.5 months (95% CI, 36.4–44.6 months), the median time to progression was 6.6 months (95% CI, 3.9–9.4 months) and the median overall survival was 22.9 months (95% CI, 17.2–28.5 months).

At a median follow-up of 40.5 months (95% CI, 36.4–44.6 months), the median time to progression was 6.6 months (95% CI, 3.9–9.4 months) and the median overall survival was 22.9 months (95% CI, 17.2–28.5 months). The time to progression in responders were 16.7 months (P < 0.0001). The Kaplan-Meier method was used to estimate overall survival and time to progression, as shown in Figure 1. At the time of present analyses, 21 patients (54%) died.

We observed no significant differences in overall response rate or survival between primary and secondary anthracycline-resistant groups. The overall survival and time to progression were higher in patients with objective response (P < 0.0001 and P = 0.05, respectively).

### Toxicity

Patients received a median of 6 cycles (range, 1–6) of docetaxel plus cisplatin. Relative dose intensities were 84% (95% CI, 73–96%) and 86% (95% CI, 75–97%) for docetaxel and cisplatin, respectively. Twenty-four patients (62%) completed 6 cycles of chemotherapy as planned; Nine patients had progression of disease while receiving treatment and 6 patients discontinued treatment because of toxicity. Treatment delay was occurred in 54 cycles (29%).

Toxicity was evaluable in all 39 patients and 189 chemotherapy cycles. Even if the most frequent hematological toxicity was neutropenia (grade 3/4 in 22% of treatment cycles), only 12 episodes of non-fatal febrile neutropenia were observed. The most commonly encountered non-hematologic toxicities were asthenia and nausea. Other toxicities are reported per patient and per cycle in Table 2. Six patients had severe toxicity precluding further treatment. No treatment-related deaths were observed.

## Discussion

Single-agent docetaxel has been considered a standard treatment for patients with anthracycline-pretreated MBC for several years. Docetaxel monotherapy often demonstrated response rates of 50% or more in this setting [17,18]. Ahn *et al*. also reported, in a similar patient population, 36% response rate and 69 weeks median survival with docetaxel 75 mg/m^2 ^every 3 weeks[19].

Platinum-based combinations also have significant activity in previously treated as well as previously untreated patients. In several small clinical trials testing the cisplatin-based regimens, response rates of 50%-83% were reported [20]. The North Central Cancer Treatment Group (NCCTG) evaluated carboplatin combined with paclitaxel as first-line chemotherapy for MBC [21]. The overall response rate was 62% and the median time to progression was 7.3 months. Clearly, platinum-based combinations are very active against MBC, but the relative degree of toxicity compared with other agents limited their use in routine clinical practice. Recently, synergism between platinum and trastuzumab, a novel monoclonal antibody directed against the protein product of the HER2/neu oncogene, awaked interest in the use of cisplatin for breast cancer [6,22].

There are relatively few completed clinical studies involving platinum-based combination chemotherapy for anthracycline-resistant MBC. Spielmann *et al*. evaluated docetaxel 75 mg/m^2 ^plus cisplatin 80 mg/m^2 ^every 3 weeks in 38 patients with anthracycline-resistant MBC, giving an objective response rate of 36% [23]. Japanese investigators performed a phase II study of docetaxel 60 mg/m^2 ^and cisplatin 80 mg/m^2 ^in patients with anthracycline-pretreated MBC [24]. They reported an overall response rate of 64%. In a phase II study of docetaxel and carboplatin, overall response rate of 61% was achieved in patients with chemotherapy-pretreated MBC [25]. Gelmon *et al*. combined biweekly paclitaxel with cisplatin and achieved a response rate of 85% with few septic events [26].

We classified "anthracycline-resistant" into primary and secondary. Ando *et al*. suggested that the status of anthracycline-resistance is important for the prediction of response to second-line treatment with docetaxel [27]. However, we could not observe significant difference in the efficacy of docetaxel plus cisplatin between primary and secondary resistant groups. The different outcomes might be due to the current situation that there have been no standard criteria defining anthracycline-resistance and often intermingled with "anthracycline-pretreated" or "anthracycline-refractory."

This study demonstrated that a combination of docetaxel plus cisplatin in a 3-week cycle was an effective and well-tolerated regimen for patients with anthracycline-resistant MBC. In this study, the use of docetaxel plus cisplatin resulted in an overall response rate of 31% and the median time to progression was 7 months. Significant improvements in the actuarial survival rate and time to progression were observed in the group of patients who achieved objective responses. The main toxicities, gastrointestinal, hematological and asthenia, were manageable with dose adjustment and supportive care. Dose intensities of more than 80% were delivered and 61% of patients completed 6 cycles of chemotherapy, which is considered acceptable and expected. We used the planned dose of cisplatin 60 mg/m^2 ^every 3 weeks, rather than 75 mg/m^2 ^cited in a phase I study [14], because we believed that tolerability of treatment is indispensable in the salvage setting in the management of solid tumors.

With the increasing use of anthracycline-based chemotherapy as adjuvant treatment, as well as first-line chemotherapy against MBC, patients are frequently exposed to high cumulative doses of anthracyclines and are therefore at risk of resistance and cardiotoxicity [28]. This combination of docetaxel with cisplatin may be particularly useful in patients previously treated with anthracyclines (but naïve to either docetaxel or cisplatin). In addition, for patients who have already had cardiac failure and have not received chemotherapy with taxanes as an adjuvant or as first-line treatment, use of docetaxel plus cisplatin is considered a better option. More recently, reports that trastuzumab has a powerful synergistic interaction with docetaxel and with cisplatin [29], have prompted evaluation of the combination of trastuzumab with docetaxel and/or platinum in the treatment of MBC [30,31].

## Conclusion

In summary, the combination of docetaxel plus cisplatin is active and safe in patients with anthracycline-resistant MBC. The activity observed in anthracycline-resistant and heavily-pretreated patients suggests relative non-cross-resistance with other drug combinations. Therefore, we hope that this study could result in a prospective trial to determine whether this activity translates into actual improvement in survival and quality of life in patients with anthracycline-resistant MBC.

## Competing interests

The author(s) declare that they have no competing interests.

## Authors' contributions

SHP collected the data, performed the statistical analysis and drafted the manuscript. EKC, SB, JHL, YDL followed the patients. DBS designed the study, followed the patients and helped with the manuscript. All authors read and approved the final manuscript.

## Pre-publication history

The pre-publication history for this paper can be accessed here:



## Figures and Tables

**Table 1:** Patient characteristics

	No.	%
Patients		
Treated	39	100.0
Evaluable for response	36	92.3
Age		
Median (years)	51.3	
Range	41.2 – 64.1	
ECOG performance status		
0	3	7.7
1	27	69.2
2	9	23.1
Site(s) of metastatic lesion(s)*		
Lung	27	69.2
Liver	9	23.1
Lymph node	15	38.5
Soft tissue	3	7.7
Bone	24	61.5
Brain	3	7.7
Response to prior anthracyclines		
Primary resistant	24	61.5
Secondary resistant	15	38.5
Interval from the cessation of last chemotherapy		
Median (months)	4.1	
Range	1.0 – 11.2	
No. of prior chemotherapy regimens		
Hormonal therapy (total)	20	51.3
1	9	23.1
2	21	53.8
3 or more	9	23.1
Characteristics of prior chemotherapy†		
CMF	24	
CAF	15	
FEC	9	
VE	21	
High-dose therapy with stem cell rescue	6	
AT	3	

* Because patients could have metastases at multiple sites, the total numbers of metastases are greater than the number of patients.

† CMF, cyclophosphamide 600 mg/m^2^, methotrexate 40 mg/m^2^, 5-fluorouracil 600 mg/m^2^, days 1 & 8, every 4 weeks; CAF, cyclophosphamide 500 mg/m^2^, doxorubicin 50 mg/m^2^, 5-fluorouracil 500 mg/m^2^, every 3 weeks; FEC, 5-fluorouracil 600 mg/m^2^, epirubicin 60 mg/m^2^, cyclophosphamide 600 mg/m^2^, every 4 weeks; VE, vinorelbine 25 mg/m^2^, days 1 & 8, epirubicin 60 mg/m^2 ^day 1, every 3 weeks; AT, doxorubicin 50 mg/m^2^, paclitaxel 175 mg/m^2^, every 3 weeks.

**Table 2:** Toxicity of chemotherapy*

	Grade 1,2		Grade 3,4	
	
	/ 39 pts	/ 189 cycles	/ 39 pts	/ 189 cycles
Febrile neutropenia			12 (31%)	12 (22%)
Neutropenia	18 (46%)	28 (15%)	15 (39%)	42 (22%)
Thrombocytopenia	18 (46%)	24 (13%)	6 (15%)	9 (5%)
Nausea/vomiting	27 (69%)	42 (22%)	24 (62%)	42 (22%)
Oral mucositis	4 (10%)	5 (3%)	3 (8%)	3 (2%)
Asthenia	12 (31%)	12 (22%)	15 (39%)	24 (13%)
Peripheral neuropathy	6 (15%)	7 (4%)	6 (15%)	9 (5%)
Hearing impairment	3 (8%)	3 (2%)		
Renal insufficiency	1 (3%)	1 (1%)		

* Others: abdominal pain(1), skin rash(1), fatigue(6)
